# A novel UCHL_3_ inhibitor, perifosine, enhances PARP inhibitor cytotoxicity through inhibition of homologous recombination-mediated DNA double strand break repair

**DOI:** 10.1038/s41419-019-1628-8

**Published:** 2019-05-21

**Authors:** Zhiwang Song, Xinyi Tu, Qin Zhou, Jinzhou Huang, Yuping Chen, Jiaqi Liu, SeungBaek Lee, Wootae Kim, Somaira Nowsheen, Kuntian Luo, Jian Yuan, Zhenkun Lou

**Affiliations:** 1 Division of Oncology Research, Mayo Clinic, Rochester, MN USA; 2 Medical Scientist Training Program, Mayo Clinic Alix School of Medicine, Mayo Clinic Graduate School of Biomedical Sciences, Rochester, MN 55905 USA; 3 Department of Molecular Pharmacology, Mayo Clinic, Rochester, MN USA

**Keywords:** Cell growth, Cancer therapeutic resistance

## Abstract

Triple-negative breast cancer (TNBC) treatment remains a great challenge for clinical practice and novel therapeutic strategies are urgently needed. UCHL3 is a deubiquitinase that is overexpressed in TNBC and correlates with poor prognosis. UCHL3 deubiquitinates RAD51 thereby promoting the recruitment of RAD51 to DNA damage sites and augmenting DNA repair. Therefore, UCHL3 overexpression can render cancer cells resistant to DNA damage inducing chemo and radiotherapy, and targeting UCHL3 can sensitize TNBC to radiation and chemotherapy. However, small molecule inhibitors of UCHL3 are yet to be identified. Here we report that perifosine, a previously reported Akt inhibitor, can inhibit UCHL3 in vitro and in vivo. We found low dose (50 nM) perifosine inhibited UCHL3 deubiquitination activity without affecting Akt activity. Furthermore, perifosine enhanced Olaparib-induced growth inhibition in TNBC cells. Mechanistically, perifosine induced RAD51 ubiquitination and blocked the RAD51-BRCA2 interaction, which in turn decreased ionizing radiation-induced foci (IRIF) of Rad51 and, thereby, homologous recombination (HR)-mediated DNA double strand break repair. In addition, combination of perifosine and Olaparib showed synergistic antitumor activity in vivo in TNBC xenograft model. Thus, our present study provides a novel therapeutic approach to optimize PARP inhibitor treatment efficiency.

## Introduction

Triple-negative breast cancer (TNBC) is an aggressive human cancer and accounts for ~15% of all breast cancer^1–3^. Since it lacks receptors, TNBC cannot be treated with HER2-targeted or hormonal therapy. Thus, therapeutic options for TNBC remain limited and conventional chemotherapy is the mainstay of TNBC treatment^4^. Coupled with the high incidence of resistance and early metastasis, the prognosis of TNBC patients remains poor^5^. Thus, it is imperative that we develop novel therapeutic strategies to manage this challenging disease.

Poly (ADP-ribose) polymerase (PARP), as a DNA nick-sensor, is required for the repair of DNA single-strand breaks (SSBs)^6^. PARP inhibitors are effective against cancer cells with defective HR-mediated DSB repair. Olaparib, a PARP1 inhibitor, was developed for the treatment of cancers with defects in DNA repair, especially tumors with BRCA mutations. It received FDA approval for the treatment of advanced ovarian cancer with defective BRCA gene and gBRCA1/2m HER2-negative metastatic breast cancer patients who previously received chemotherapy in adjuvant, neoadjuvant, or metastatic settings^7–9^. However, BRCA1/2 mutations account for only 2–3% of all breast cancers^10^, and PARP inhibitors failed to improve prognosis over chemotherapy alone in a phase III trial^11^.

We previously found that UCHL3 promotes HR by causing deubiquitination of RAD51 and promoting the binding of RAD51 with BRCA2^12^. UCHL3 depletion sensitizes breast cancer cells to radiation and chemotherapy, while overexpression of UCHL3 renders cells resistant to these therapies^12^. Interestingly, UCHL3 is overexpressed in TNBC and higher UCHL3 expression correlates with poor prognosis^12^. However, specific inhibitors of UCHL3 are not yet available. Here, we found that low dose perifosine, a previously identified AKT inhibitor, inhibits UCHL3 deubiquitination activity without affecting AKT activity. Moreover, perifosine strongly suppresses HR-mediated DSB repair by increasing RAD51 ubiquitination and inhibiting Rad51 function. Finally, perifosine significantly enhances Olaparib-induced antitumor effect. Collectively, our work provides a novel strategy to enhance PARP inhibitor anticancer effect in TNBC.

## Methods

### Cytotoxicity and colony formation assays

Cells were seeded into 96-well plates. Twenty-four hour later, cells were treated with drugs at different concentrations. After 10 days, cells were washed with PBS, fixed with methanol, and stained. Finally, the colony numbers were counted.

### Immunofluorescence for nuclear foci

Cells were seeded on coverslips, treated, and then washed with PBS. Cells were fixed with 3% paraformaldehyde, permeabilized with 0.5% Triton-X, blocked using 5% goat serum, and incubated with anti-RAD51, BRCA1, or BRCA2 antibody. Next, cells were incubated with secondary antibodies and cell nuclei were counterstained with DAPI. Finally, the signals were examined by confocal microscopy.

### Cell cycle analysis

Treated cells were fixed with 70% ethanol at −20 °C overnight and stained with propidium iodide (PI) containing RNAse for 30 min in the dark. Cell cycle was analyzed using FACS and ModFit LT software.

### HR repair assay

MDA-MB-231 cells stably transfected with the HR reporter DR-GFP (MDA-MB-231-DR-GFP) were treated with or without 50 nM perifosine for 24 h. Then, the cells were transfected with pCBA-I-Sce-I. Forty-eight hour later, GFP expression was analyzed by flow cytometry.

### CRISPR/Cas9 knockout

As described in our previous paper^12^, we cloned the sequence of small guide RNA (sgUCHL3 5′-GCCGCTGGAGGCCAATCCCGAGG-3′) into the vector LentiCRISPR-V2-puro. MDA-MB-231 cells were infected with Lenti-UCHL3-sgRNA-puro. Then, stable clones were selected using 2 μg/mL puromycin, and single colonies were obtained through serial dilution and amplification. Finally, immunoblotting and DNA sequencing were used to identify the colonies.

### Denatured deubiquitination assay in vivo and deubiquitination assay in vitro

As described in our previous paper^12^, for the deubiquitination assay in vivo, control MDA-MB-231 cells and UCHL3 knockout MDA-MB-231 cells were treated with 50 nM perifosine for 24 h, then collected, lysed, and centrifuged. The cell extracts were used to perform deubiquitination assay and immunoprecipitation experiment. For the deubiquitination assay in vitro, we first purified ubiquitinated proteins from cell extracts with nickel (His) beads under denaturing conditions. Then, the Ub-RAD51 and UCHL3 wild-type (WT) proteins were purified according to standard protocol. Ubiquitinated proteins were incubated with recombinant UCHL3 in a deubiquitination buffer for 4 h at 30 °C.

### In vivo antitumor study

1 × 10^6^ MDA-MB-231 cells were subcutaneously injected into the flanks of 5-week old female nude mice. Tumor volumes were evaluated every 4 days and calculated using the formula: V = (L × W^2^)/2 (V, volume; L, length; and W, width). When tumors reached mean volumes of 120–180 mm^3^, they were randomly divided into four groups (1–4) with eight mice per group. Group 1 was intraperitoneally injected with 0.1 ml PBS as control; Group 2 received 1 mg/kg of perifosine intraperitoneally t.i.d; Group 3 received 50 mg/kg of olaparib t.i.d by intraperitoneal injection, and Group 4 received their combination.

### Statistics

All the data are presented as mean ± SD. GraphPad Prism software (GraphPad Inc. USA) was used to perform statistical analysis. Two tailed Student’s *t*-test or χ^2^ test was adopted to evaluate statistical significance between groups, and *P* < 0.05 was considered to be a significant difference. The statistical significance in all figures are represented by: *P < 0.05; **P < 0.01, ***P < 0.001, and n.s. no significance.

## Results

### Perifosine sensitizes TNBC cells to Olaparib

**Fig. 1:**
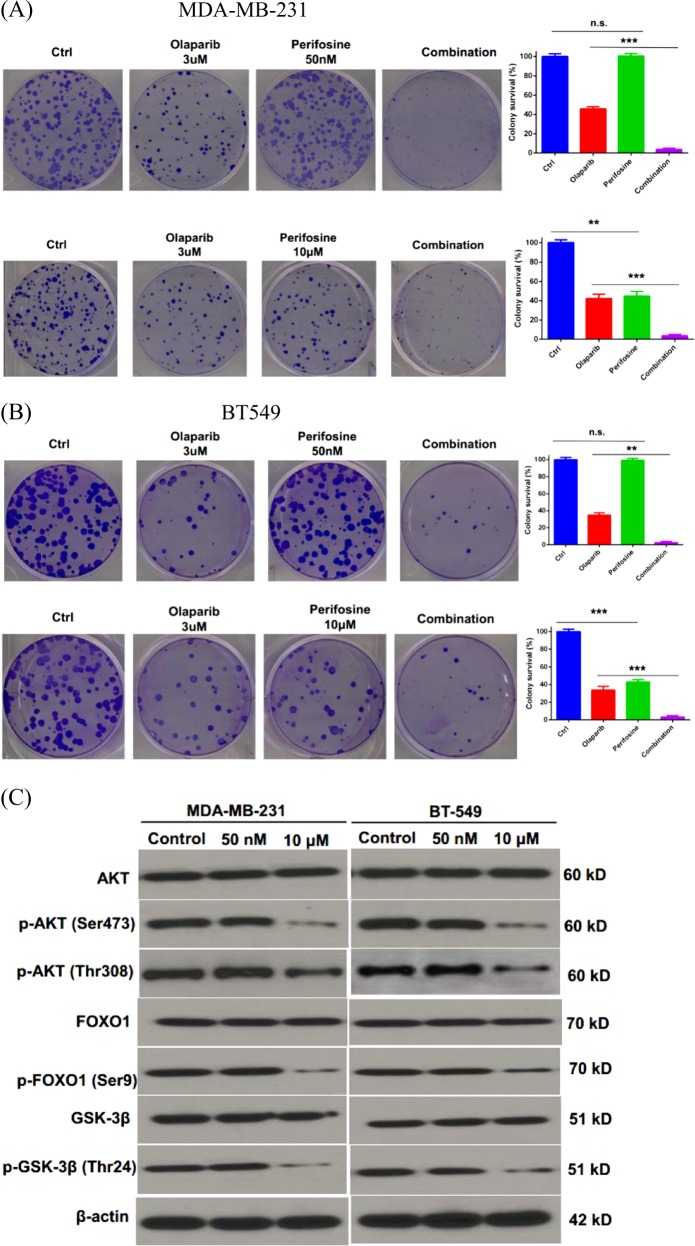
Perifosine enhances the killing of TNBC cells by Olaparib. **a**, **b** Colony formation assay of MDA-MB-231 (**a**) or BT-549 (**b**) cells after treatment with 3 µM Olaparib, 50 nM, or 10 μM perifosine or their combination. **c** Western blot analysis of Akt, p-Akt, FOXO1, p-FOXO1, GSK-3β, and p-GSK-3β after 50 nM or 10 μM perifosine treatment for 24 h. β-actin was used as a loading control. **P < 0.01, ***P < 0.001, and n.s. no significance

AKT, a serine/threonine kinase, functions as a central regulator of multiple cellular processes including cell growth, proliferation, metabolism, motility, survival, and apoptosis. We were interested in testing the role of AKT in HR-mediated DNA repair, which is highly related to chemoresistance in TNBC. We utilized perifosine, an AKT inhibitor, to treat the TNBC cell lines, MDA-MB-231 and BT549. As shown in Fig. 1a–c, high dose of perifosine (10 µM) inhibited cancer cell proliferation and phosphorylation of AKT and its downstream targets, FOXO1 and GSK-3β^13,14^. However, low dose of perifosine (50 nM) did not affect cancer cell proliferation and the phosphorylation status of AKT, FOXO1, and GSK-3β (Fig. 1a–c). Interestingly, we found that low dose of perifosine treatment (50 nM) led to synthetic lethality with Olaparib in MDA-MB-231 and BT549 cells (Fig. 1a, b). Taken together, these results suggested that perifosine may target a novel substrate, which in turn enhances PARP inhibitor (PARPi) efficiency in TNBC cells.

### Perifosine potentiates Olaparib-induced DNA damage

**Fig. 2:**
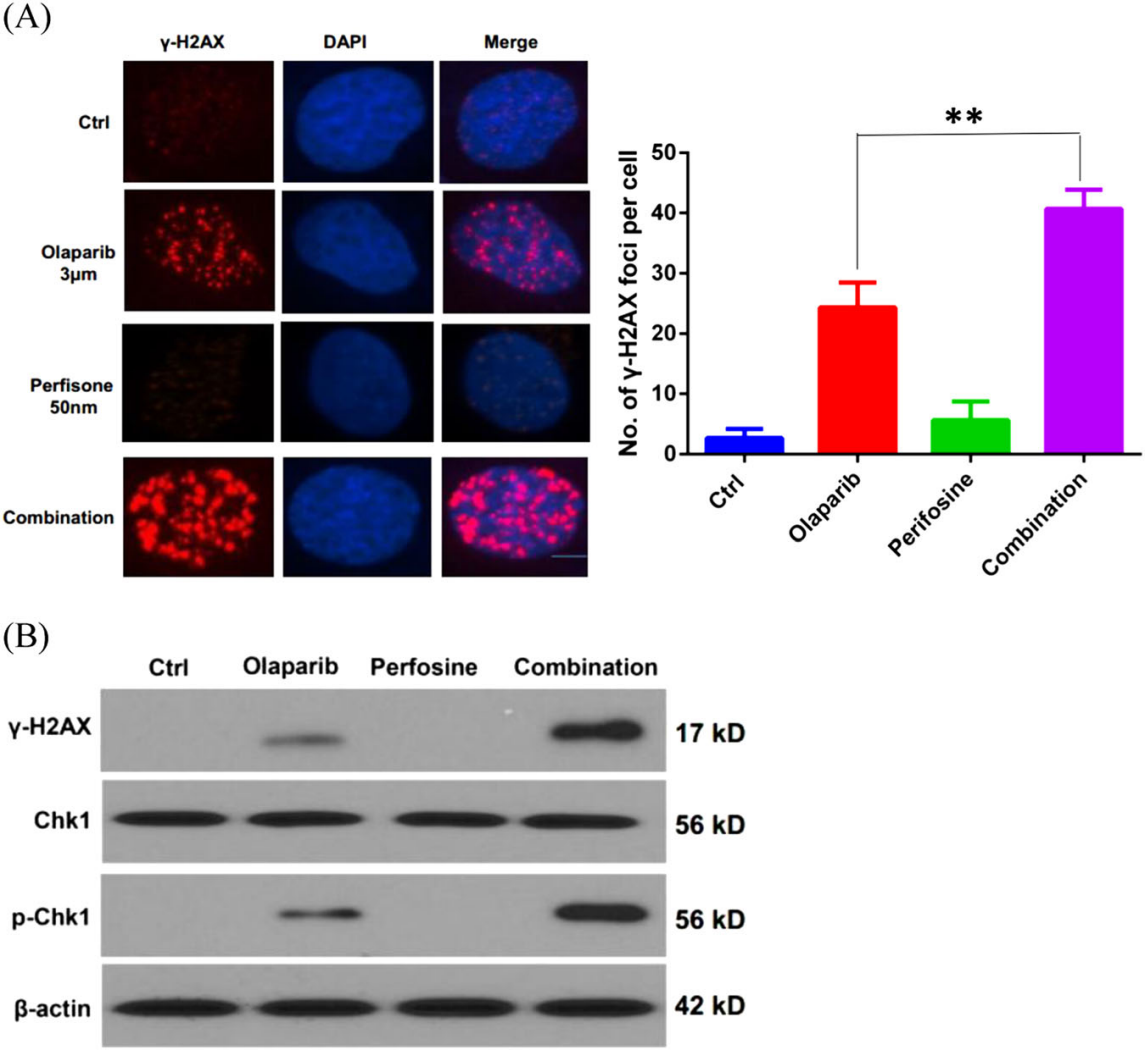
Perifosine enhances Olaparib-induced DNA damage. **a** γ-H2AX staining in MDA-MB-231 cells treated with 3 µM of olaparib, 50 nM perifosine, or their combination. **b** Western blot analysis of γ-H2AX, Chk1, and p-Chk1 after indicated treatment. β-actin was used as a loading control. **P < 0.01

To evaluate whether the synergistic activity induced by combination of perifosine and Olaparib results from increased DNA damage, γH2AX staining, a marker of DNA damage, was used to assess the level of DNA damage. As shown in Fig. 2a, Olaparib treatment caused γH2AX focus formation. Low dose of perifosine treatment alone did not significantly induce γH2AX foci. However, the γH2AX foci formation was dramatically increased in cells with combination treatment of perifosine and Olaparib compared to Olaparib treatment alone. Furthermore, western blot analysis showed that the combination treatment markedly increased γH2AX and p-Chk1 levels, although perifosine itself did not induce DNA damage signaling (Fig. 2b). Collectively, these results indicated that perifosine significantly increases the DNA damage induced by Olaparib.

### Perifosine impairs HR repair

**Fig. 3:**
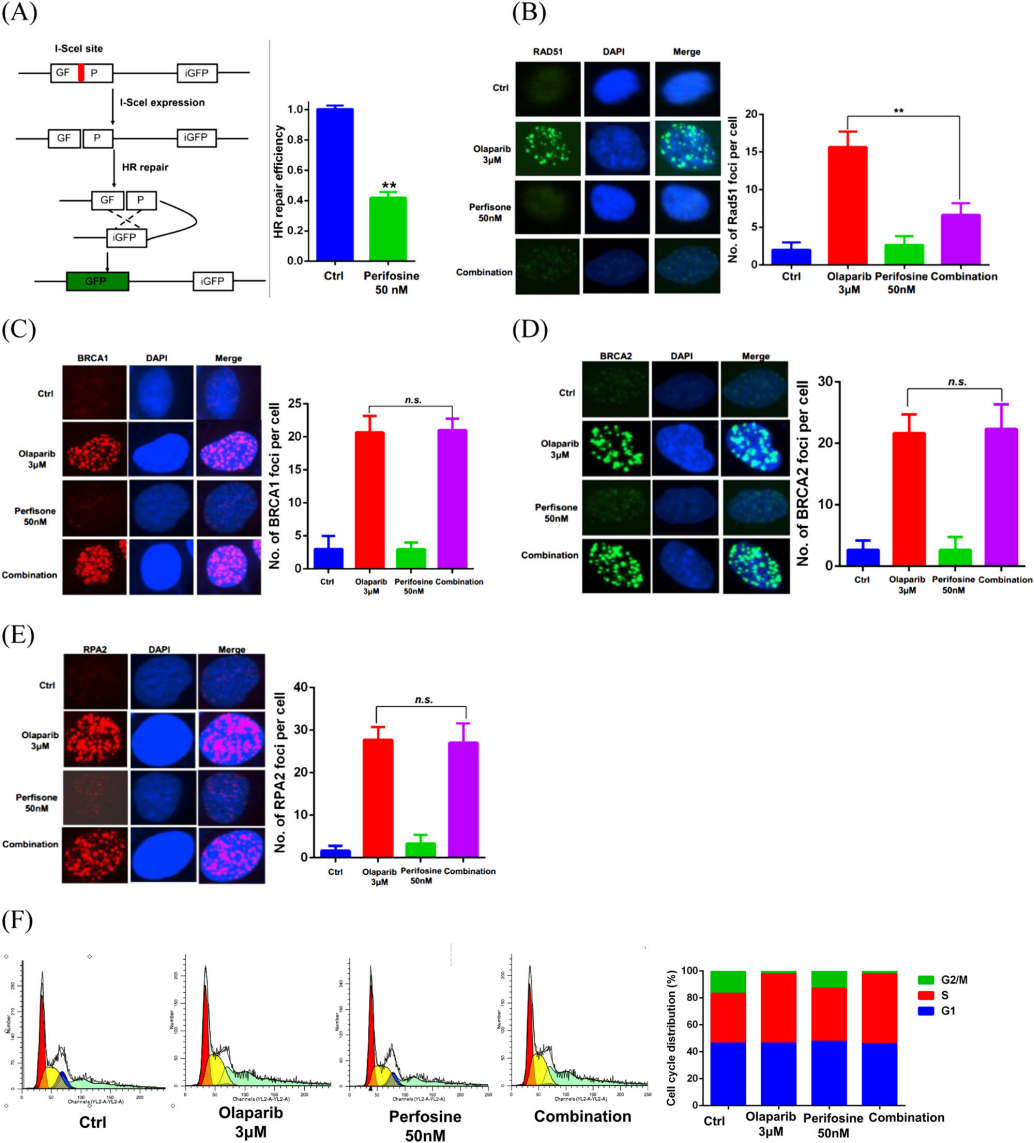
Perifosine reduces HR repair capability. **a** (Left) Schematic diagram of HR reporter system. (Right) Analysis of HR repair activity in MDA-MB-231 cells treated with 50 nM of perifosine. **b**–**e** Analysis of RAD51 foci (**b**), BRCA1 foci (**c**), BRCA2 foci (**d**), or RPA2 foci (**e**) in MDA-MB-231 cells after exposure to indicated treatment for 24 h. **f** Perifosine did not change cell cycle distribution of MDA-MB-231 cells. **P < 0.01, n.s. no significance

Since previous studies established that HR defects sensitize cells to PARP inhibitors, we were interested in examining the effect of perifosine treatment on HR-mediated DSB repair. As shown in Fig. 3a–e, low dose perifosine treatment significantly decreased HR repair in cells, while significantly decreased Olaparib-induced RAD51 foci, although it did not affect Olaparib-induced BRCA1, BRCA2, and RPA2 foci formation. In addition, as shown in Fig. 3f, the cell cycle distribution was not significantly different between Olaparib alone and combination treatments, suggesting that perifosine-induced HR deficiency is not related to a change in cell cycle distribution. Taken together, these data suggested that low dose of perifosine impairs HR repair by inhibiting RAD51 foci formation.

### Perifosine regulates RAD51 ubiquitination by inhibiting UCHL3

**Fig. 4:**
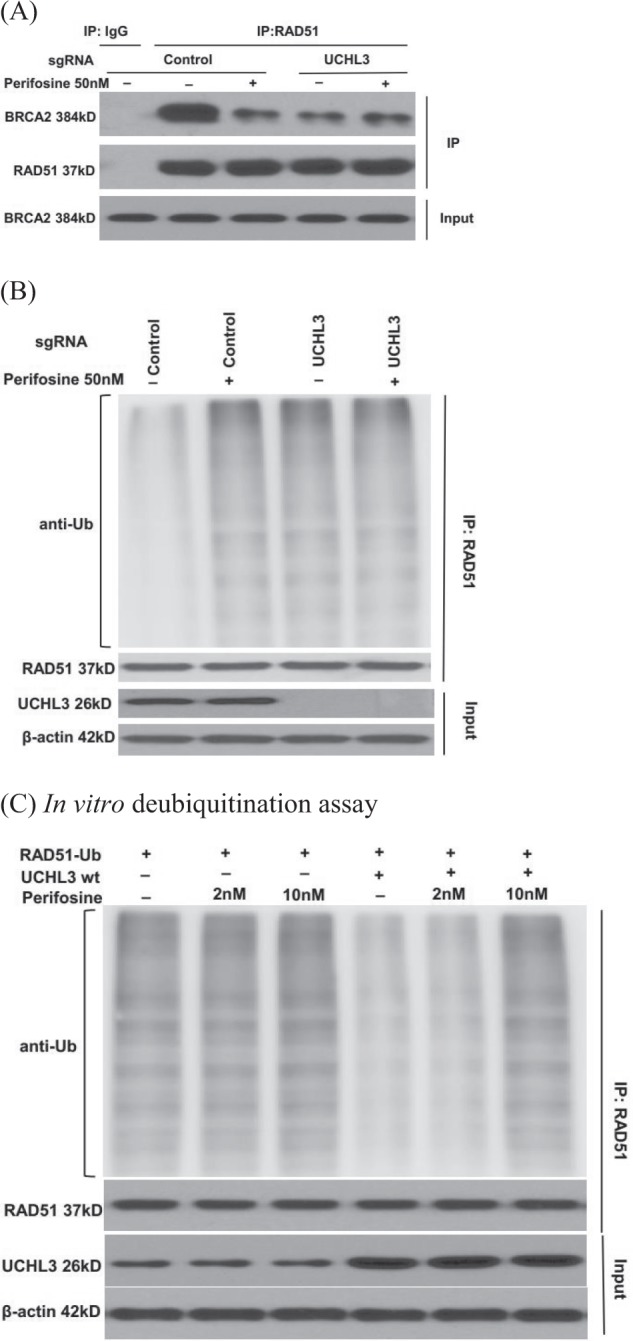
Perifosine regulates RAD51 ubiquitination by inhibiting UCHL3. **a** Control and UCHL3 knockout MDA-MB-231 cells were treated with perifosine and lysates were used for immunoprecipitation and western blot as indicated. **b** Examination of Rad51 ubiquitination in cells treated with perifosine. **c** In vitro deubiquitination analysis of UCHL3 in the presence or absence of perifosine

RAD51, a strand-exchange protein, is a central player in HR. Previous studies showed that BRCA2 directly binds to RAD51 and recruits it to double stand break sites following DNA damage. Our lab has previously reported that the deubiquitination enzyme UCHL3 deubiquitinates RAD51, which in turn facilities RAD51-BRCA2 interaction and RAD51 foci formation^12^. We next examined whether perifosine decreases RAD51 foci by blocking the BRCA2-RAD51 interaction. As shown in Fig. 4a, perifosine treatment dramatically decreased the interaction between BRCA2 and RAD51. Interestingly, perifosine treatment did not further decrease the BRCA2-RAD51 interaction in UCHL3 knockout cells, suggesting that perifosine may regulate BRCA2-RAD51 interaction through UCHL3. In addition, we used DLD-1 cell line (BRCA2 deficient cell line) to perform colony formation assay. As shown in Supplementary Fig. 1, low dose of perifosine treatment (50 nM) failed to cause synthetic lethality with Olaparib in DLD-1 cells, which further proved that the mechanism is through disrupted interaction between BRCA2 and RAD51. Since UCHL3 deubiquitinates RAD51, which in turn regulates the BRCA2-RAD51 interaction, we next tested whether perifosine regulates UCHL3 deubiquitination activity. As shown in Fig. 4b, perifosine treatment dramatically induced RAD51 ubiquitination, similar to UCHL3 depletion. Furthermore, perifosine had no further effect on RAD51 ubiquitination in UCHL3 knockout cells, suggesting that perifosine regulates RAD51 deubiquitination through UCHL3. This also led us to hypothesize that perifosine might directly inhibit UCHL3. To test this hypothesis, we performed in vitro deubiquitination assays. As shown in Fig. 4c, UCHL3 directly deubiquitinated RAD51. However, treatment with 10 nM perifosine was able to inhibit UCHL3-mediated Rad51 deubiquitination in vitro (comparing lanes 1, 4, and 6). In the absence of UCHL3, perifosine did not affect RAD51 deubiquitination (comparing lanes 1, 2, and 3). Taken together, these data suggested that the AKT inhibitor perifosine has an off-target effect at low dose by targeting UCHL3, which in turn impairs RAD51 foci formation and HR repair.

### Combination therapy with perifosine and Olaparib synergistically inhibits TNBC growth in vivo

**Fig. 5:**
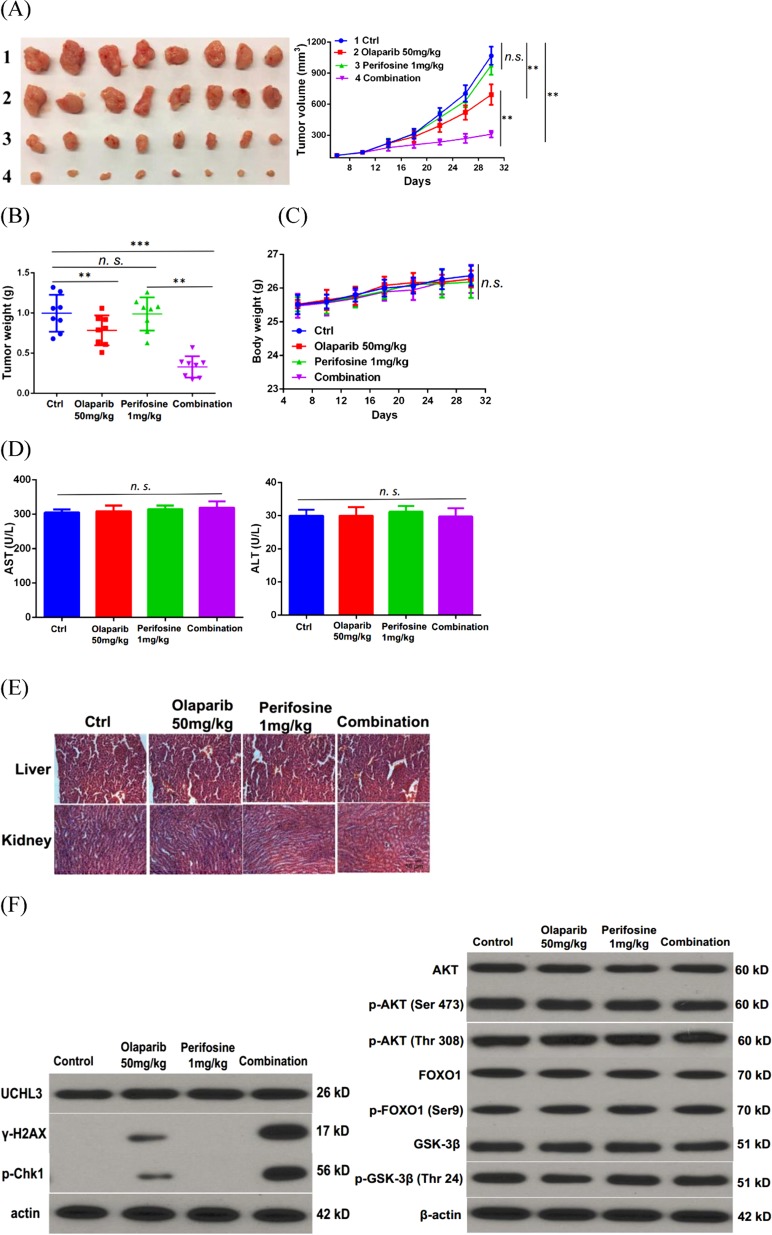
Combination of perifosine and Olaparib synergistically represses triple-negative breast cancer growth in vivo. **a**, **b** Mice bearing MDA-MB-231 xenografts were treated with vehicle control, perifosine (1 mg/kg, t.i.d.), olaparib (50 mg/kg/, t.i.d), or their combination. Tumor growth curve (**a**) and tumor weight (**b**) are shown. Data presented as mean ± s.d. of eight mice per group. **c** Body weight of mice treated with control (Ctrl), 1 mg/kg perifosine, 50 mg/kg Olaparib, or their combination. **d** Serum analysis including alanine aminotransferase (ALT) and aspartate aminotransferase (AST) of mice after various treatment. Data presented as mean ± s.d. of three replicates. **e** Hematoxylin and eosin staining of liver and kidney organs after various treatments (×20). **f** Western blot analysis of γ-H2AX, Chk1, p-Chk1, Akt, p-Akt, FOXO1, p-FOXO1, GSK-3β, and p-GSK-3β levels in tumors of xenograft model. β-actin was used as a loading control. **P < 0.01, ***P < 0.001, and n.s. no significance

Since low dose perifosine treatment impairs HR and sensitizes TNBC cells to PARPi in vitro, we next examined whether combination treatment with low dose of perifosine (1 mg/kg) and Olaparib (50 mg/kg) can kill TNBC more efficiently in xenograft models. As shown in Fig. 5a, b, low dose of perifosine treatment alone did not show obvious antitumor effect. However, the combination treatment inhibited TNBC cancer cell growth more efficiently compared to Olaparib treatment alone.

Furthermore, our data suggested that the combination treatment was well tolerated without obvious weight loss (Fig. 5c) and obvious increase in ALT and AST levels (Fig. 5d). Histopathological analysis further demonstrated no evidence of toxicity in normal tissue (Fig. 5e). Moreover, western blot results further proved that low dose of perifosine treatment increased DNA damage caused by olaparib without affecting Akt pathway in vivo (Fig. 5f). Taken together, these results suggested that combination treatment with low dose of perifosine and Olaparib may be a novel strategy to overcome chemoresistance in TNBC.

## Discussion

PARP inhibitors have shown the most promising effects on treatment of BRCA1/2-deficient individuals including TNBC patients^15,16^. However, most TNBC patients are BRCA1/2-proficient for whom olaparib as a single agent treatment provided a rather limited therapeutic opportunity^17^. Thus, combination of PARP inhibitor with additional agents that inhibit HR repair may be an effective approach to enhance activity of PARP inhibitor in TNBC that are Olaparib resistant.

Here we showed that perifosine greatly sensitizes TNBC cells to Olaparib treatment, and combination treatment of perifosine and Olaparib induces synergistic antitumor effect both in vitro and in vivo. Therefore, our study provides a novel therapeutic strategy for PARP inhibitor-mediated TNBC treatment.

Perifosine (octadecyl-(1,1-dimethyl-piperdinio-4-yl)-phosphate), the first synthetic and oral bioactive alkyl phospholipid, has shown anticancer effect in a wide range of cancer cells. The biological mechanism by which perifosine exerts tumor-inhibitory activity remains to be fully elucidated, but it is widely reported that the anticancer function of perifosine is mainly due to inhibition of both constitutive and inducible phosphorylation of AKT and blocked AKT activation^18,19^. Furthermore, perifosine has been previously shown to enhance the effect of radiotherapy and chemotherapy in addition to antitumor effects^20–22^. In line with the above observation, our study suggests that perifosine enhances Olaparib-induced antitumor effect in vitro and in vivo. Importantly, we used a low dose of perifosine, which neither affects AKT pathway nor shows antitumor effect alone. This low dose has the potential to significantly decrease side effects related to chemotherapy.

We further explored the potential mechanism by which perifosine enhanced the antitumor effect of Olaparib, and we found that perifosine inhibits UCHL3 and UCHL3-mediated deubiquitination of RAD51, thus disrupting HR repair function. RAD51, a central protein in HR, directs homologous pairing and DNA strand exchange that is crucial for HR repair^23^. The ubiquitination of RAD51 blocks its interaction with BRCA2 and impairs HR repair^12,24,25^. Therefore, perifosine potentiates the activity of Olaparib by compromising RAD51 function and suppressing HR repair.

## Conclusions

In summary, we illustrated that perifosine enhances the therapeutic efficacy of Olaparib in human TNBC cell lines as well as xenograft mouse model. Moreover, our results indicated that perifosine is a UCHL3 inhibitor that promotes ubiquitination of RAD51 and thereby disrupts HR repair. Perifosine therefore impairs HR repair efficacy and promotes accumulation of DNA damage caused by Olaparib treatment and subsequently induces cell death. Combination of PARP inhibitor and agents that inhibit HR was universally accepted as a promising strategy to potentiate the cytotoxicity of PARP inhibitors. Our findings support the clinical development of perifosine and PARP inhibitor combination for the treatment on human TNBC.

## Supplementary information

Supplementary Materials

Supplementary figure legends
