# Supplementary material for: A novel UCHL3 inhibitor, perifosine, enhances PARP inhibitor cytotoxicity through inhibition of homologous recombination-mediated DNA double strand break repair
Source: Cell Death Dis. 2019 May 21;10(6):398. doi: 10.1038/s41419-019-1628-8 (PMC6529448; doi:10.1038/s41419-019-1628-8)

**Cell culture and antibodies**

All the used cell lines were purchased from American Type Culture Collection (ATCC, USA). Antibodies against Akt, phospho-Akt (Ser473), phospho-Akt (Thr308), FOXO1, phospho- FOXO1 (Ser 9), GSK-3β, phospho- GSK-3β (Ther 24), Chk1, and phospho-Chk1 (Ser345) were purchased from Cell Signaling Inc. Anti-UCHL3 (12384-1-AP) antibody was purchased from ProteinTech. Anti-BRCA1 (D9) and Anti-Ub (P4D1) antibodies were purchased from Santa Cruz Biotechnology. Anti-RAD51 (N1C2) was purchased from GeneTex. Anti-γH2AX (05-636) and Anti-BRCA2 (A303-435) antibodies were purchased from Millipore. Secondary antibodies against mouse (sc-2748) and rabbit (sc-2750) were purchased from Santa Cruz. Anti-β-actin antibody was purchased from Sigma.

Supplementary Figure 1

DLD-1


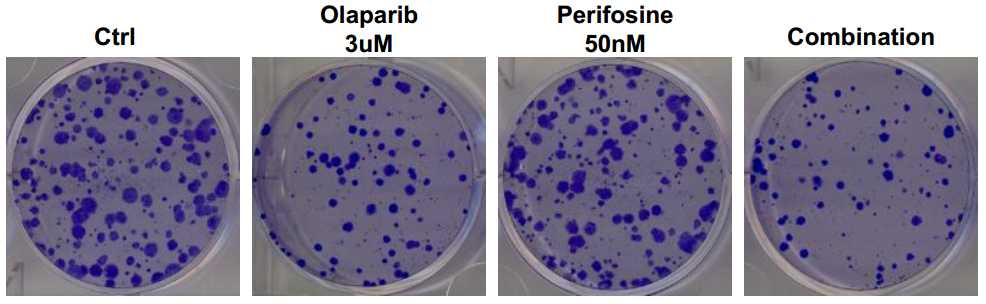

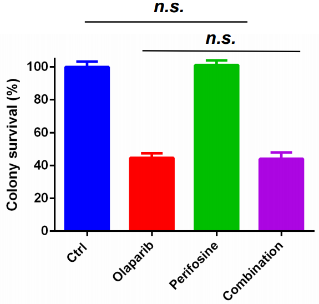


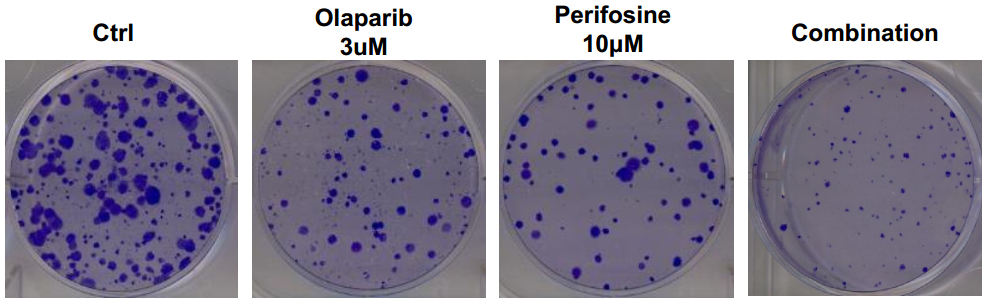

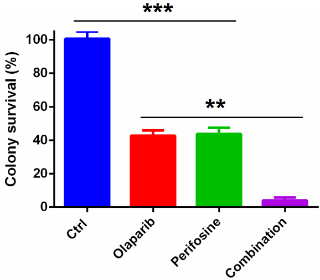

Supplement: Supplementary file 1 — Supplementary Materials [file 41419_2019_1628_MOESM1_ESM.doc]
