# Supplementary material for: A novel UCHL3 inhibitor, perifosine, enhances PARP inhibitor cytotoxicity through inhibition of homologous recombination-mediated DNA double strand break repair
Source: Cell Death Dis. 2019 May 21;10(6):398. doi: 10.1038/s41419-019-1628-8 (PMC6529448; doi:10.1038/s41419-019-1628-8)
Supplement: Supplementary file 2 — Supplementary figure legends [file 41419_2019_1628_MOESM2_ESM.docx]

Supplementary Figure legends:

**Supplementary Figure 1** Perifosine does not affect the response of BRCA2 deficient cells to Olaparib. Colony formation assay of DLD-1 cells after treatment with 3 µM Olaparib, 50 nM or 10 μM perifosine or their combination. ***P* < 0.01, ****P* < 0.001, and *n.s.* no significance.
